# Book Review: Microbial Inoculants in Sustainable Agricultural Productivity- Vol. II: Functional Application

**DOI:** 10.3389/fmicb.2016.02105

**Published:** 2016-12-23

**Authors:** Jay Shankar Singh, Sumit Koushal, Arun Kumar, Shobhit R. Vimal, Vijai K. Gupta

**Affiliations:** ^1^Department of Environmental Microbiology, Babasaheb Bhimrao Ambedkar (Central) UniversityLucknow, India; ^2^Molecular Glyco-biotechnology Group, Discipline of Biochemistry, School of Natural Sciences, National University of Ireland Galway (NUI)Galway, Ireland

**Keywords:** agriculture, cyanobacteria, inoculants, microbes, mycorrhiza

Ecologically sustainable agricultural practices are essential to ensure food security, and efficient agriculturally beneficial microbes (microbial inoculants) are playing potential role in sustainable crop production due to their immense plant growth promoting attributes, better adaptability to survival under stresses, and other uses that result in attenuating the pesticides/fertilizers use in agriculture. However, the unpredictable biogenic and abiogenic soil factors that determine the nature and magnitude of the microbial inoculants responses and survival after their delivery in the field conditions remain unresolved.

Soil microbes via several processes play indispensible roles in the supply of valuable nutrients to crop plants (Bashan et al., 2014). The presence of beneficial microbial communities in the rhizosphere minimizes the susceptibility to crop diseases (Singh, 2015). Because of ability to produce plant growth promoting and other molecules from secondary metabolism, beneficial microbes are widely used as commercial bio-inoculants (Singh et al., 2016a). However, a great diversity of valuable microbial inoculants continues to be revealed, and little is known about to the potential applications of new efficient microbial formulations that have been described.

The book *Microbial Inoculants in Sustainable Agricultural Productivity Vol. II Functional Applications* essentially addresses the field usage of microbial agents (biofertilizers, biostimulants, biopesticides) for boosting agriculture sustainability. In this volume, a total of 19 chapters have been distributed over 308 pages. It contains the relevant topics contributed by the well-known researchers from different universities and institutes. Entire chapters in different subject areas contributed by the leading authors can be grouped into four parts. The first part (chapter 1–11) highlights the use of bio-inoculants in management of crop plant stresses. This section provide satisfactory information about diverse group of microbes (rhizobia, cyanobacteria, actinomycetes, mycorrhiza, endophytes, etc.) that have been developed as microbial inoculants with beneficial functions at different levels and many chapters have touched on commercial production for applications in field conditions for farmers' benefits. Recently, plant growth promoting rhizobacteria (PGPRs) and cyanobacteria have gained attention for their indispensible role in restoration ecology and sustainable agriculture (Singh and Strong, 2016; Singh et al., 2016b). Therefore, selection of such efficient microbial strains with well-defined PGP mechanisms can be exploited in development of bio-fertilizer/bio-pesticide inoculants for achieving consistent and economical results under field conditions. Furthermore, these microbial inoculants with tested results can be considered as integrated nutrient management system to sustain agricultural productivity with no adverse environmental impact. Though, these bio-agents play a significant role in soil nutrient management, the knowledge about their roles, functioning and the mechanism of action is not completely understood under field conditions. Therefore, the performance of microbial inoculants under field conditions may be governed by more than one mechanism/factor and is a complex phenomenon that needs comprehensive study (Logue et al., 2015).

The second part of the book (chapter 12–15) is exclusively dedicated to explain about formulations, applications and delivery systems of microbial inoculants. Microbial bio-formulations commercially available and their mode of application in the field along with conventional methods of the delivery systems such as microbigation, seed bio-priming, seed encapsulation, fluid drilling, and consortia are also discussed. This volume confirmed that the common microbial agents used for the purpose of bio-inoculants production should have higher viable microbial count in field conditions, extended shelf life, efficiency, resistance to biotic and abiotic stresses, competence to indigenous soil micro-flora, affordability to farmers, and ability to create overall positive impacts on agriculture and environment.

The third part of the book (chapter 16–18) emphasizes about nanoparticles mediated novel technologies geared toward crop health. Nanoparticles based smart delivery system like nanosensors, nanofertilizers, nanopesticides, and nanoformulations to enhance agricultural health is also pointed out. Finally the last section of the book (chapter 19) highlights mainly on regulation and registration of bioinoculants. Registration requirement of bio-pesticides is mandatory to ensure safety to human health, benign effects to non-target organisms, and the environment. Although, in certain countries implemented regulations and framework indicated that use of bio-pesticides in agriculture is safe for human health, there are also some challenges and technical problems that need to be addressed through more effective policies and scientific approaches to enhance quality and safety of bio-pesticide.

The book lacks studies on impact of delivered microbial bio-inoculants on native microbial community structure and their interaction with soil and plants. Also, it is outside the purview of the book to review the methodologies which have been applied for monitoring the performance of introduced microbial inoculants in field conditions. It is evident from the investigations that the systems involving plant growth promotion by bio-agents include, in addition to the direct microbial consequences, their communication by signaling molecules with indigenous micro-flora, and the resulting impact on soil functioning. As most plant-inoculants interactions are as yet unexplored and high-throughput genomic and proteomic tools are limited, we can only predict that plant metabolite-mediated microbial inoculants differentiations could be general strategies of the plant-inoculants associations (Maróti and Kondorosi, 2014). Therefore, there is need to evaluate crucially such non-target effects of microbial inoculants at genomic, transcriptomic, and proteomic level, and to authenticate such results before their delivery in the natural field conditions. The information available in the book are quantitative with no attempt made to evaluate the impact of microbial inoculants on factors involved in N dynamics, an important driving force of soil, microbes, and plant sustenance. Nowadays bio-formulations (microbes or their metabolites) application has been considered as promising tools for sustainable crop production. Inoculation of beneficial microbes along with their metabolites may be a potential option, proving to be more valuable with multifarious roles and consequently indicating the new avenue to develop the bio-formulations for safe agriculture (Arora and Mishra, 2016). However, the eco-friendly viable bio-formulations development at commercial level and constraints associated with them are not discussed in this volume.

In conclusion, this volume offered detailed information on mass production of efficient microbial inoculants from laboratory to industrial/commercial level, intellectual property rights required, registration processes, bio-safety and bio-security concerns, quality control, and their legal authenticity. Based on the above merits, we recommend this book to stakeholders engaged in working on microbial bioinoculants.

## Author contributions

All authors listed, have made substantial, direct and intellectual contribution to the work, and approved it for publication.

### Conflict of interest statement

VG discloses a conflict of interest with Harikesh Bahadur Singh, one of the Editors of the reviewed volume, and with Braj Raj Singh who has contributed a chapter in the reviewed volume. VG has collaborated with Harikesh Bahadur Singh and Braj Raj Singh for the purposes of writing scientific papers that have been published. VG hereby confirms that this relationship did not influence what has been written in this book review.
